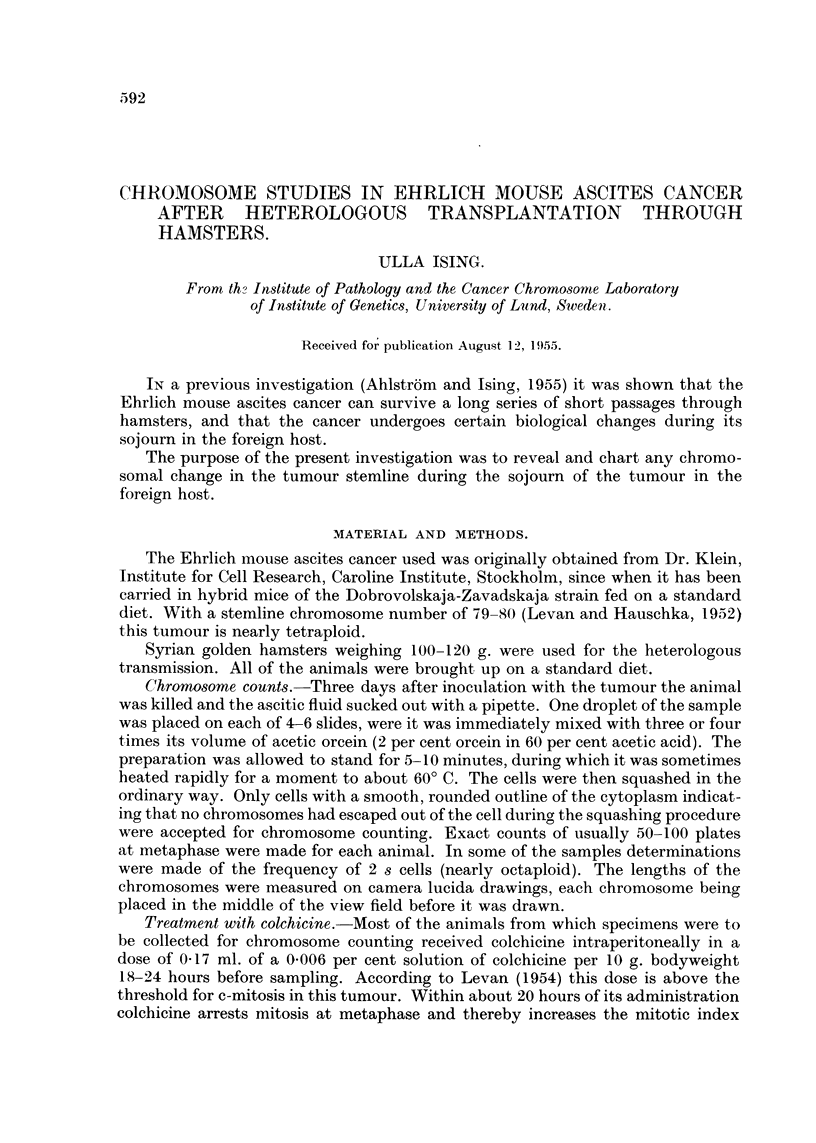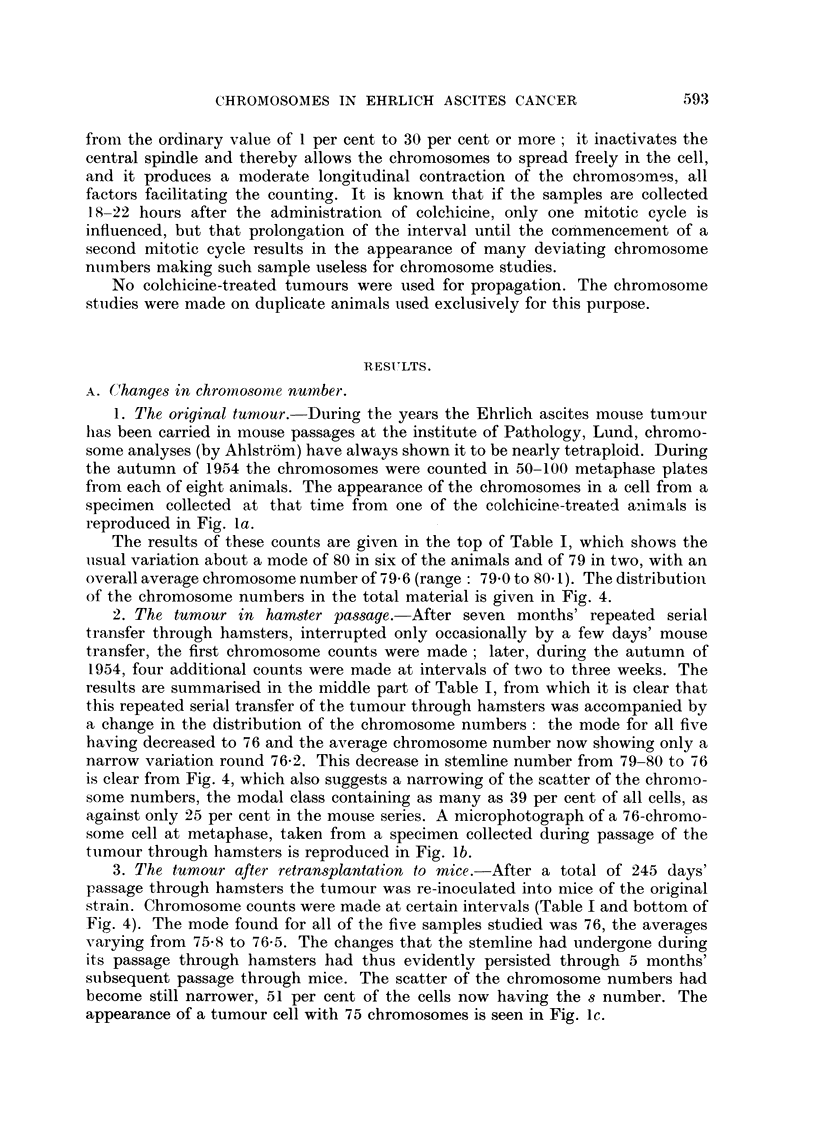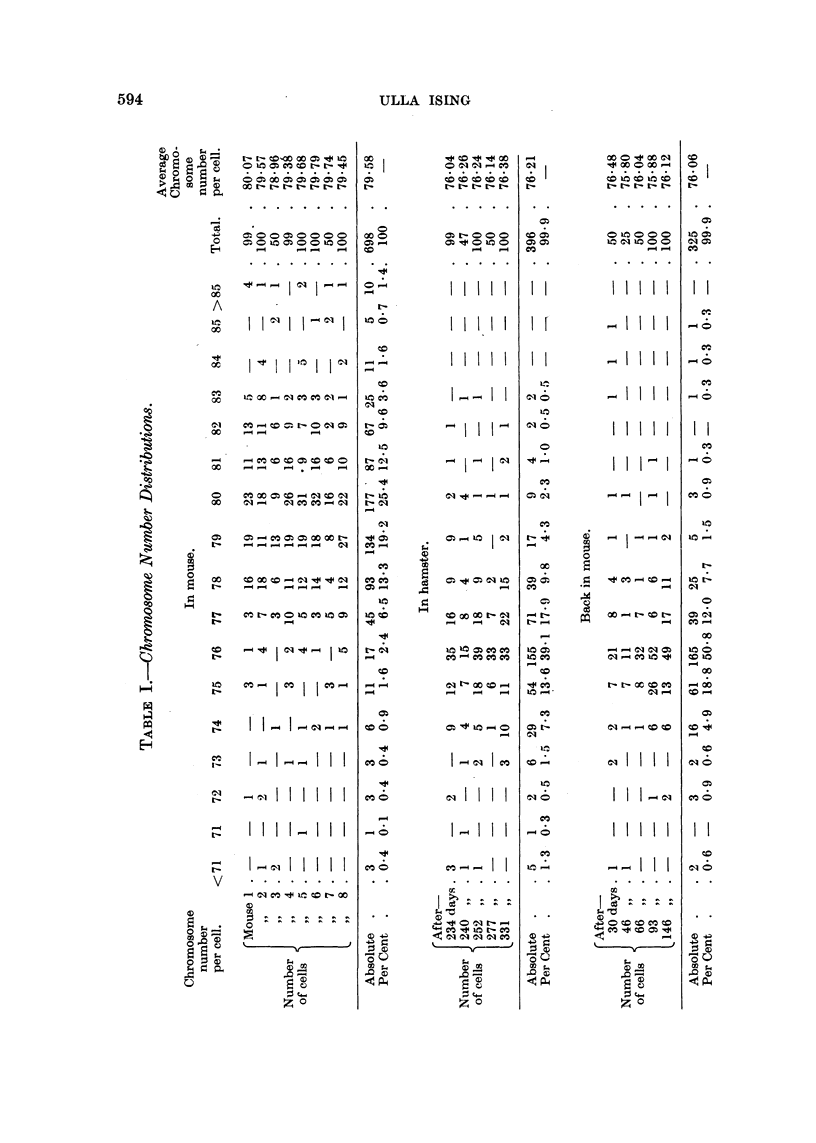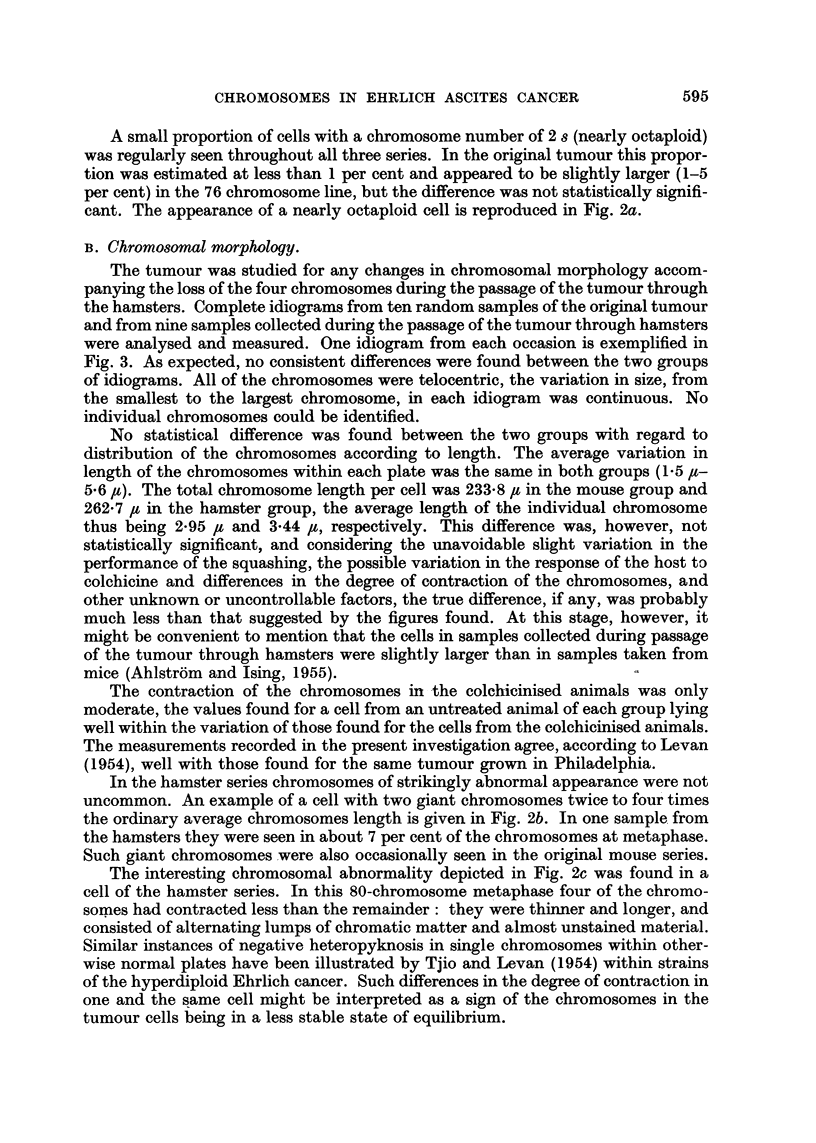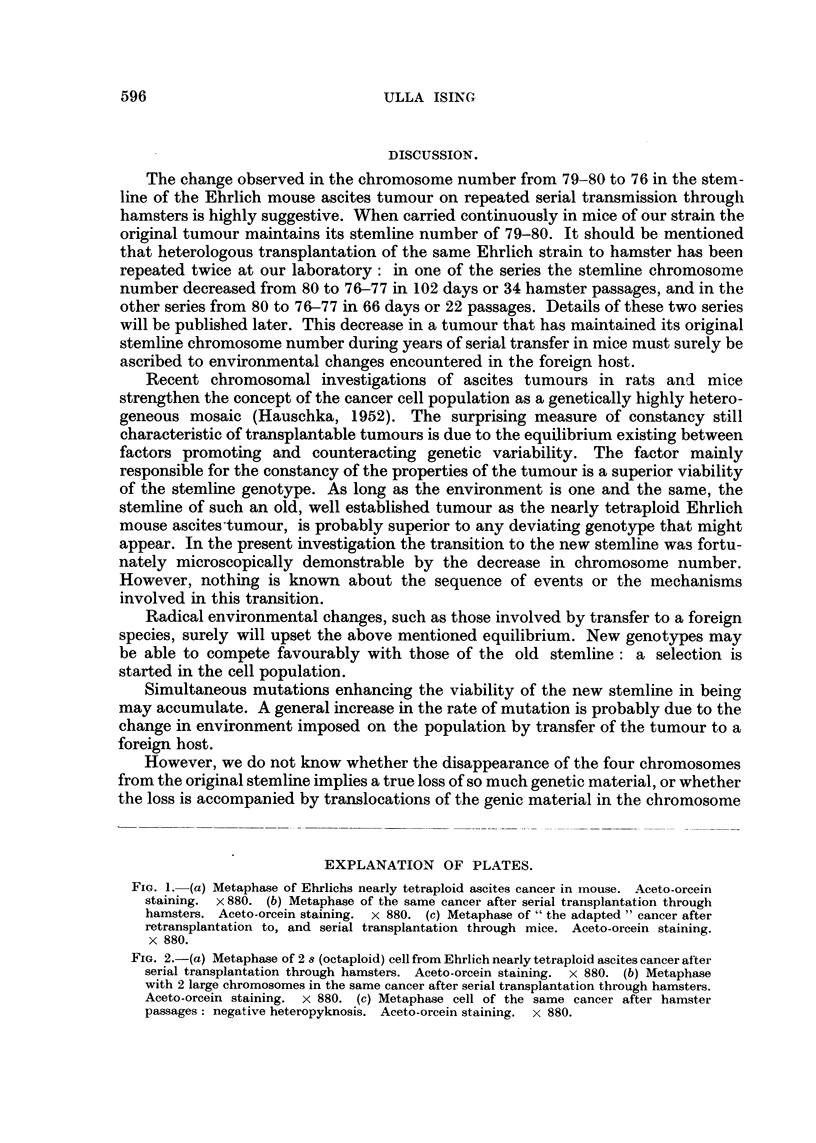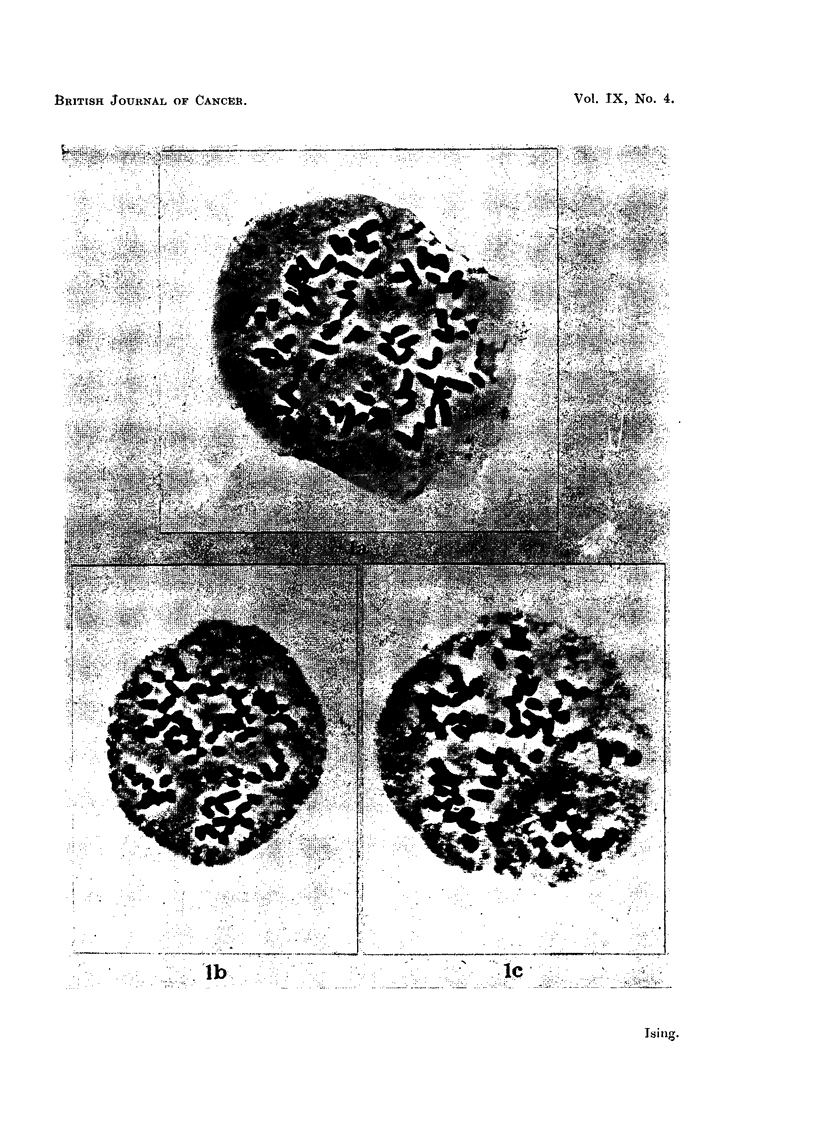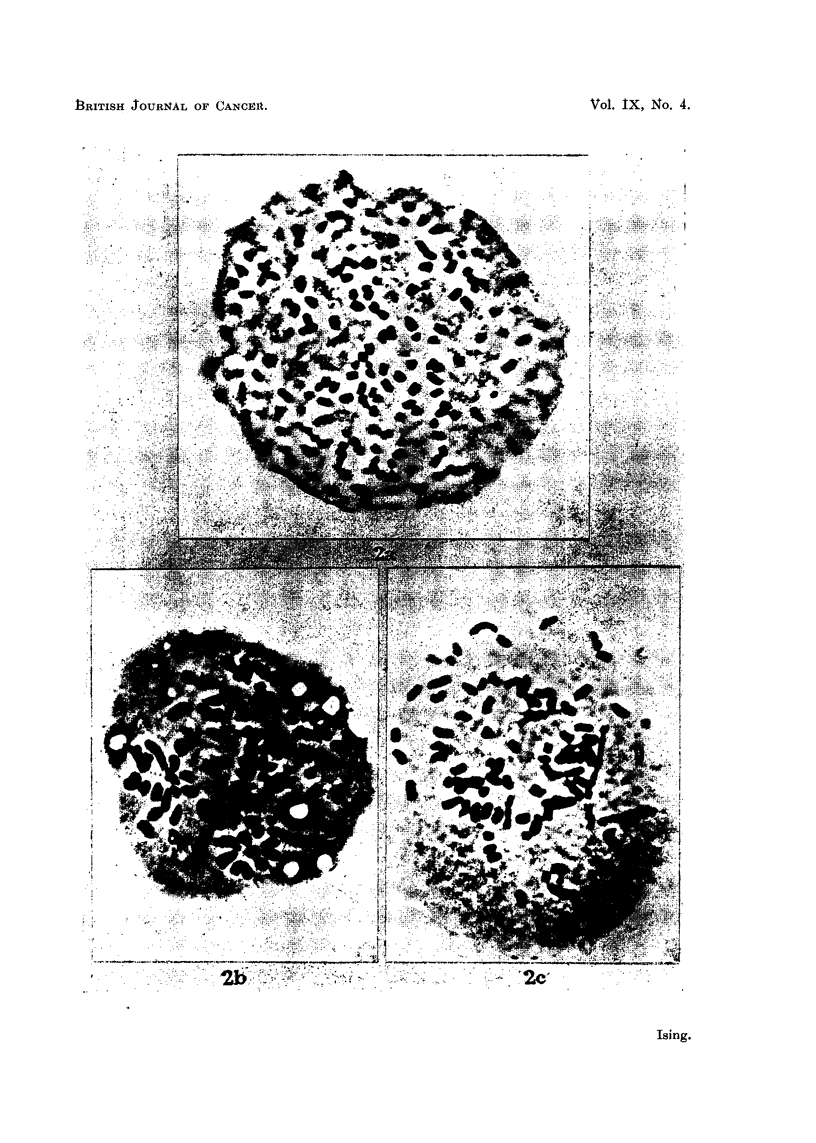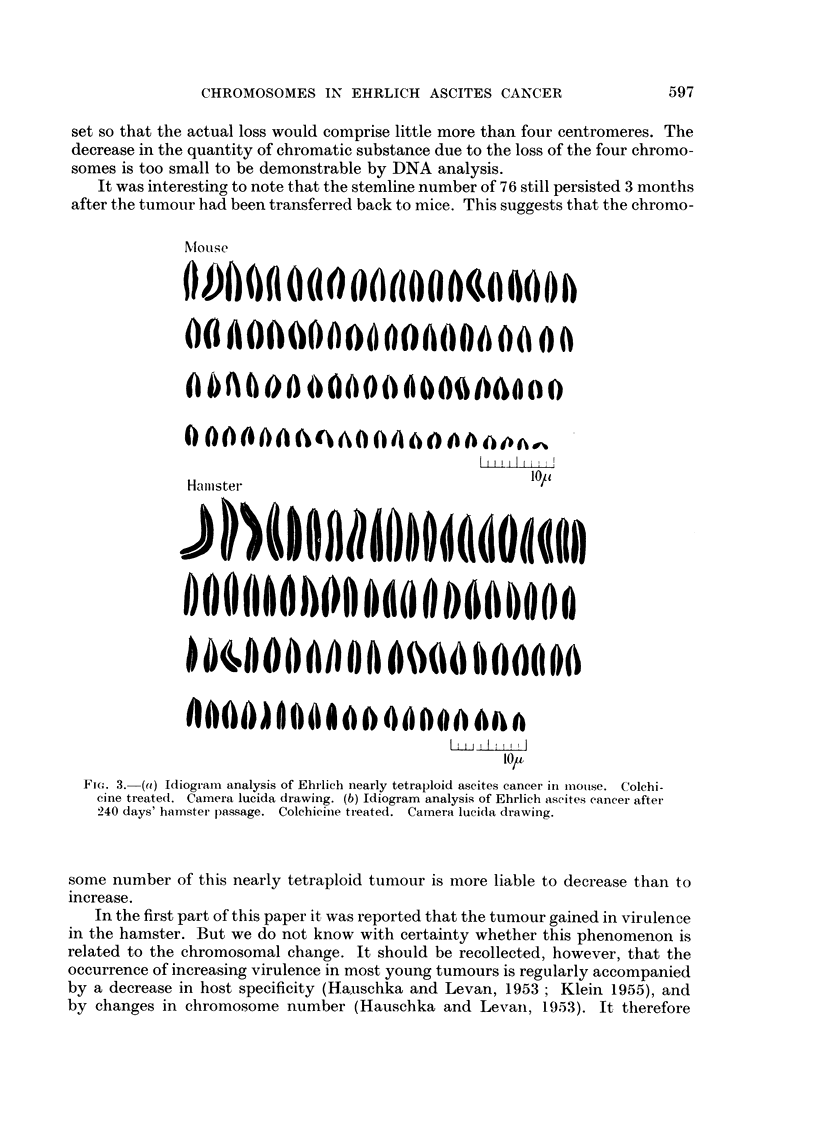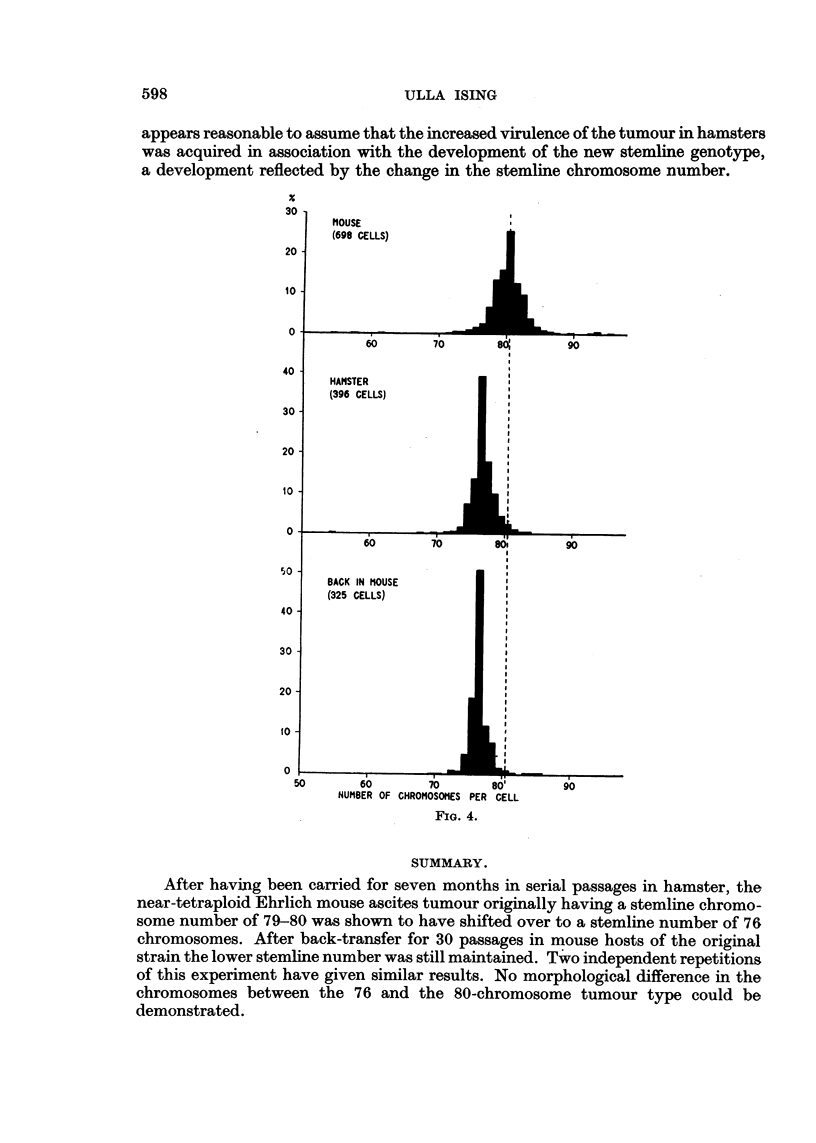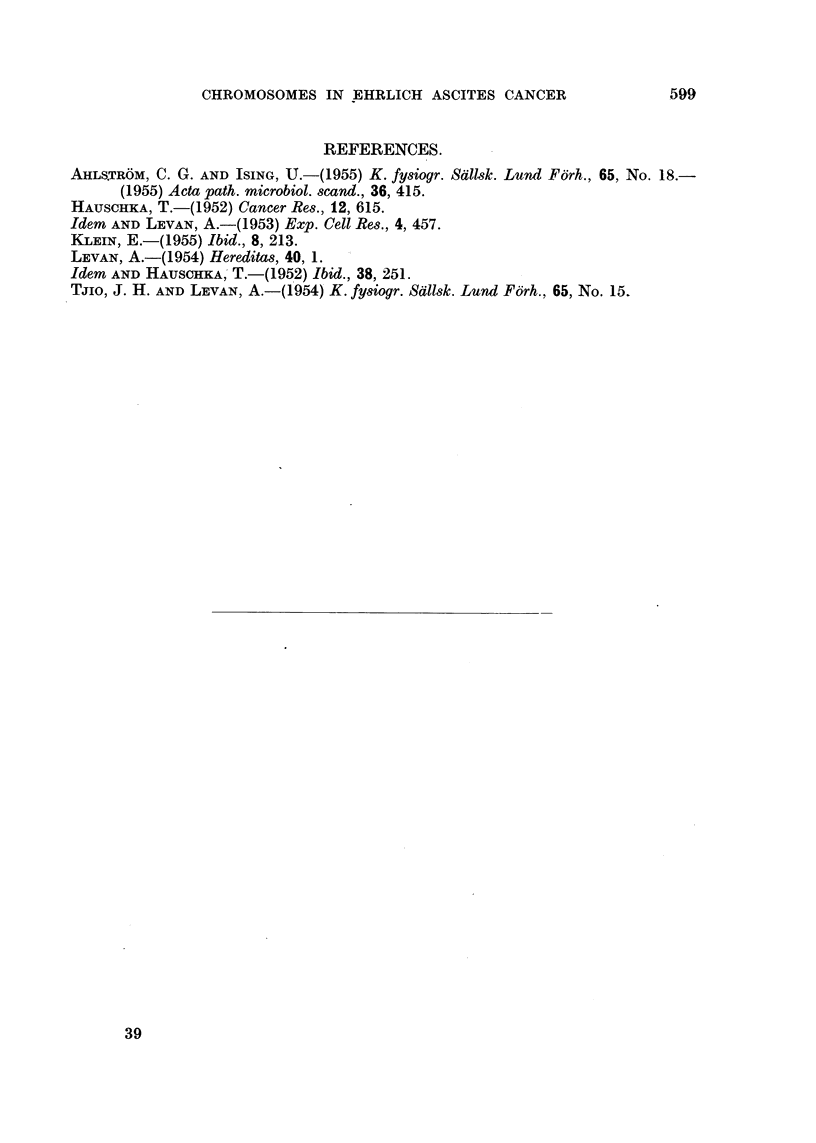# Chromosome Studies in Ehrlich Mouse Ascites Cancer after Heterologous Transplantation through Hamsters

**DOI:** 10.1038/bjc.1955.60

**Published:** 1955-12

**Authors:** Ulla Ising

## Abstract

**Images:**


### CHROMOSOME STUDIES IN EHRLICH MOUSE ASCITES CANCER AFTER HETEROLOGOUS TRANSPLANTATION THROUGH HAMSTERS.

ULLA ISING.

*From the Institute of Pathology and the Cancer Chromosome Laboratory of Institute of Genetics, University of Lund, Sweden.*

Received for publication August 12, 1955.

IN a previous investigation (Ahlström and Ising, 1955) it was shown that the Ehrlich mouse ascites cancer can survive a long series of short passages through hamsters, and that the cancer undergoes certain biological changes during its sojourn in the foreign host.

The purpose of the present investigation was to reveal and chart any chromosomal change in the tumour stemline during the sojourn of the tumour in the foreign host.

### MATERIAL AND METHODS.

The Ehrlich mouse ascites cancer used was originally obtained from Dr. Klein, Institute for Cell Research, Caroline Institute, Stockholm, since when it has been carried in hybrid mice of the Dobrovolskaja-Zavadskaja strain fed on a standard diet. With a stemline chromosome number of 79–80 (Levan and Hauschka, 1952) this tumour is nearly tetraploid.

Syrian golden hamsters weighing 100–120 g. were used for the heterologous transmission. All of the animals were brought up on a standard diet.

*Chromosome counts.*—Three days after inoculation with the tumour the animal was killed and the ascitic fluid sucked out with a pipette. One droplet of the sample was placed on each of 4–6 slides, were it was immediately mixed with three or four times its volume of acetic orcein (2 per cent orcein in 60 per cent acetic acid). The preparation was allowed to stand for 5–10 minutes, during which it was sometimes heated rapidly for a moment to about 60° C. The cells were then squashed in the ordinary way. Only cells with a smooth, rounded outline of the cytoplasm indicating that no chromosomes had escaped out of the cell during the squashing procedure were accepted for chromosome counting. Exact counts of usually 50–100 plates at metaphase were made for each animal. In some of the samples determinations were made of the frequency of 2 *s* cells (nearly octaploid). The lengths of the chromosomes were measured on camera lucida drawings, each chromosome being placed in the middle of the view field before it was drawn.

*Treatment with colchicine.*—Most of the animals from which specimens were to be collected for chromosome counting received colchicine intraperitoneally in a dose of 0·17 ml. of a 0·006 per cent solution of colchicine per 10 g. bodyweight 18–24 hours before sampling. According to Levan (1954) this dose is above the threshold for c-mitosis in this tumour. Within about 20 hours of its administration colchicine arrests mitosis at metaphase and thereby increases the mitotic index from the ordinary value of 1 per cent to 30 per cent or more ; it inactivates the central spindle and thereby allows the chromosomes to spread freely in the cell, and it produces a moderate longitudinal contraction of the chromosomes, all factors facilitating the counting. It is known that if the samples are collected 18–22 hours after the administration of colchicine, only one mitotic cycle is influenced, but that prolongation of the interval until the commencement of a second mitotic cycle results in the appearance of many deviating chromosome numbers making such sample useless for chromosome studies.

No colchicine-treated tumours were used for propagation. The chromosome studies were made on duplicate animals used exclusively for this purpose.

### RESULTS.

A. *Changes in chromosome number.*

1. *The original tumour.*—During the years the Ehrlich ascites mouse tumour has been carried in mouse passages at the institute of Pathology, Lund, chromosome analyses (by Ahlström) have always shown it to be nearly tetraploid. During the autumn of 1954 the chromosomes were counted in 50–100 metaphase plates from each of eight animals. The appearance of the chromosomes in a cell from a specimen collected at that time from one of the colchicine-treated animals is reproduced in Fig. 1*a*.

The results of these counts are given in the top of Table I, which shows the usual variation about a mode of 80 in six of the animals and of 79 in two, with an overall average chromosome number of 79·6 (range : 79·0 to 80·1). The distribution of the chromosome numbers in the total material is given in Fig. 4.

2. *The tumour in hamster passage.*—After seven months' repeated serial transfer through hamsters, interrupted only occasionally by a few days' mouse transfer, the first chromosome counts were made ; later, during the autumn of 1954, four additional counts were made at intervals of two to three weeks. The results are summarised in the middle part of Table I, from which it is clear that this repeated serial transfer of the tumour through hamsters was accompanied by a change in the distribution of the chromosome numbers : the mode for all five having decreased to 76 and the average chromosome number now showing only a narrow variation round 76·2. This decrease in stemline number from 79–80 to 76 is clear from Fig. 4, which also suggests a narrowing of the scatter of the chromosome numbers, the modal class containing as many as 39 per cent of all cells, as against only 25 per cent in the mouse series. A microphotograph of a 76-chromosome cell at metaphase, taken from a specimen collected during passage of the tumour through hamsters is reproduced in Fig. 1*b*.

3. *The tumour after retransplantation to mice.*—After a total of 245 days' passage through hamsters the tumour was re-inoculated into mice of the original strain. Chromosome counts were made at certain intervals (Table I and bottom of Fig. 4). The mode found for all of the five samples studied was 76, the averages varying from 75·8 to 76·5. The changes that the stemline had undergone during its passage through hamsters had thus evidently persisted through 5 months' subsequent passage through mice. The scatter of the chromosome numbers had become still narrower, 51 per cent of the cells now having the *s* number. The appearance of a tumour cell with 75 chromosomes is seen in Fig. 1*c*.

TABLE I.—*Chromosome Number Distributions.*

| Chromosome number per cell. | <71 | 71 | 72 | 73 | 74 | 75 | 76 | 77 | 78 | 79 | 80 | 81 | 82 | 83 | 84 | 85 | >85 | Total. | Average Chromosome number per cell. |
|---|---|---|---|---|---|---|---|---|---|---|---|---|---|---|---|---|---|---|---|
| | | | | | | | | | In mouse. | | | | | | | | | | |
| Number of cells: Mouse 1 | — | — | 1 | — | — | 3 | 1 | 3 | 16 | 19 | 23 | 11 | 13 | 5 | — | — | 4 | 99 | 80·07 |
| ,, 2 | 1 | — | 2 | 1 | — | 1 | 4 | 7 | 18 | 11 | 18 | 13 | 11 | 8 | 4 | — | 1 | 100 | 79·57 |
| ,, 3 | 2 | — | — | — | 1 | — | — | 3 | 6 | 13 | 9 | 6 | 6 | 1 | — | 2 | 1 | 50 | 78·96 |
| ,, 4 | — | — | — | 1 | — | 3 | 2 | 10 | 11 | 19 | 26 | 16 | 9 | 2 | — | — | — | 99 | 79·38 |
| ,, 5 | — | 1 | — | 1 | 1 | — | 4 | 5 | 12 | 19 | 31 | 9 | 7 | 3 | 5 | — | 2 | 100 | 79·68 |
| ,, 6 | — | — | — | — | 2 | — | 1 | 3 | 14 | 18 | 32 | 16 | 10 | 3 | — | 1 | — | 100 | 79·79 |
| ,, 7 | — | — | — | — | 1 | 3 | — | 5 | 4 | 8 | 16 | 6 | 2 | 2 | — | 2 | 1 | 50 | 79·74 |
| ,, 8 | — | — | — | — | 1 | 1 | 5 | 9 | 12 | 27 | 22 | 10 | 9 | 1 | 2 | — | 1 | 100 | 79·45 |
| Absolute | 3 | 1 | 3 | 3 | 6 | 11 | 17 | 45 | 93 | 134 | 177 | 87 | 67 | 25 | 11 | 5 | 10 | 698 | 79·58 |
| Per Cent | 0·4 | 0·1 | 0·4 | 0·4 | 0·9 | 1·6 | 2·4 | 6·5 | 13·3 | 19·2 | 25·4 | 12·5 | 9·6 | 3·6 | 1·6 | 0·7 | 1·4 | 100 | — |
| | | | | | | | | | In hamster. | | | | | | | | | | |
| Number of cells: After— 234 days | 3 | — | 2 | — | 9 | 12 | 35 | 16 | 9 | 9 | 2 | 1 | 1 | — | — | — | — | 99 | 76·04 |
| 240 ,, | 1 | 1 | — | 1 | 4 | 7 | 15 | 8 | 4 | 1 | 4 | — | — | 1 | — | — | — | 47 | 76·26 |
| 252 ,, | 1 | — | — | 2 | 5 | 18 | 39 | 18 | 9 | 5 | 1 | 1 | — | 1 | — | — | — | 100 | 76·24 |
| 277 ,, | — | — | — | — | 1 | 6 | 33 | 7 | 2 | — | 1 | — | — | — | — | — | — | 50 | 76·14 |
| 331 ,, | — | — | — | 3 | 10 | 11 | 33 | 22 | 15 | 2 | 1 | 2 | 1 | — | — | — | — | 100 | 76·38 |
| Absolute | 5 | 1 | 2 | 6 | 29 | 54 | 155 | 71 | 39 | 17 | 9 | 4 | 2 | 2 | — | — | — | 396 | 76·21 |
| Per Cent | 1·3 | 0·3 | 0·5 | 1·5 | 7·3 | 13·6 | 39·1 | 17·9 | 9·8 | 4·3 | 2·3 | 1·0 | 0·5 | 0·5 | — | — | — | 99·9 | — |
| | | | | | | | | | Back in mouse. | | | | | | | | | | |
| Number of cells: After— 30 days | 1 | — | — | 2 | 2 | 7 | 21 | 8 | 4 | 1 | 1 | — | — | 1 | 1 | 1 | — | 50 | 76·48 |
| 46 ,, | 1 | — | — | — | 1 | 7 | 11 | 1 | 3 | — | 1 | — | — | — | — | — | — | 25 | 75·80 |
| 66 ,, | — | — | — | — | 1 | 8 | 32 | 7 | 1 | 1 | — | — | — | — | — | — | — | 50 | 76·04 |
| 93 ,, | — | — | 1 | — | 6 | 26 | 52 | 6 | 6 | 1 | 1 | 1 | — | — | — | — | — | 100 | 75·88 |
| 146 ,, | — | — | 2 | — | 6 | 13 | 49 | 17 | 11 | 2 | — | — | — | — | — | — | — | 100 | 76·12 |
| Absolute | 2 | — | 3 | 2 | 16 | 61 | 165 | 39 | 25 | 5 | 3 | 1 | — | 1 | 1 | 1 | — | 325 | 76·06 |
| Per Cent | 0·6 | — | 0·9 | 0·6 | 4·9 | 18·8 | 50·8 | 12·0 | 7·7 | 1·5 | 0·9 | 0·3 | — | 0·3 | 0·3 | 0·3 | — | 99·9 | — |

A small proportion of cells with a chromosome number of 2 *s* (nearly octaploid) was regularly seen throughout all three series. In the original tumour this proportion was estimated at less than 1 per cent and appeared to be slightly larger (1–5 per cent) in the 76 chromosome line, but the difference was not statistically significant. The appearance of a nearly octaploid cell is reproduced in Fig. 2*a*.

B. *Chromosomal morphology.*

The tumour was studied for any changes in chromosomal morphology accompanying the loss of the four chromosomes during the passage of the tumour through the hamsters. Complete idiograms from ten random samples of the original tumour and from nine samples collected during the passage of the tumour through hamsters were analysed and measured. One idiogram from each occasion is exemplified in Fig. 3. As expected, no consistent differences were found between the two groups of idiograms. All of the chromosomes were telocentric, the variation in size, from the smallest to the largest chromosome, in each idiogram was continuous. No individual chromosomes could be identified.

No statistical difference was found between the two groups with regard to distribution of the chromosomes according to length. The average variation in length of the chromosomes within each plate was the same in both groups (1·5 $\mu$–5·6 $\mu$). The total chromosome length per cell was 233·8 $\mu$ in the mouse group and 262·7 $\mu$ in the hamster group, the average length of the individual chromosome thus being 2·95 $\mu$ and 3·44 $\mu$, respectively. This difference was, however, not statistically significant, and considering the unavoidable slight variation in the performance of the squashing, the possible variation in the response of the host to colchicine and differences in the degree of contraction of the chromosomes, and other unknown or uncontrollable factors, the true difference, if any, was probably much less than that suggested by the figures found. At this stage, however, it might be convenient to mention that the cells in samples collected during passage of the tumour through hamsters were slightly larger than in samples taken from mice (Ahlström and Ising, 1955).

The contraction of the chromosomes in the colchicinised animals was only moderate, the values found for a cell from an untreated animal of each group lying well within the variation of those found for the cells from the colchicinised animals. The measurements recorded in the present investigation agree, according to Levan (1954), well with those found for the same tumour grown in Philadelphia.

In the hamster series chromosomes of strikingly abnormal appearance were not uncommon. An example of a cell with two giant chromosomes twice to four times the ordinary average chromosomes length is given in Fig. 2*b*. In one sample from the hamsters they were seen in about 7 per cent of the chromosomes at metaphase. Such giant chromosomes were also occasionally seen in the original mouse series.

The interesting chromosomal abnormality depicted in Fig. 2*c* was found in a cell of the hamster series. In this 80-chromosome metaphase four of the chromosomes had contracted less than the remainder : they were thinner and longer, and consisted of alternating lumps of chromatic matter and almost unstained material. Similar instances of negative heteropyknosis in single chromosomes within otherwise normal plates have been illustrated by Tjio and Levan (1954) within strains of the hyperdiploid Ehrlich cancer. Such differences in the degree of contraction in one and the same cell might be interpreted as a sign of the chromosomes in the tumour cells being in a less stable state of equilibrium.

### DISCUSSION.

The change observed in the chromosome number from 79–80 to 76 in the stemline of the Ehrlich mouse ascites tumour on repeated serial transmission through hamsters is highly suggestive. When carried continuously in mice of our strain the original tumour maintains its stemline number of 79–80. It should be mentioned that heterologous transplantation of the same Ehrlich strain to hamster has been repeated twice at our laboratory : in one of the series the stemline chromosome number decreased from 80 to 76–77 in 102 days or 34 hamster passages, and in the other series from 80 to 76–77 in 66 days or 22 passages. Details of these two series will be published later. This decrease in a tumour that has maintained its original stemline chromosome number during years of serial transfer in mice must surely be ascribed to environmental changes encountered in the foreign host.

Recent chromosomal investigations of ascites tumours in rats and mice strengthen the concept of the cancer cell population as a genetically highly heterogeneous mosaic (Hauschka, 1952). The surprising measure of constancy still characteristic of transplantable tumours is due to the equilibrium existing between factors promoting and counteracting genetic variability. The factor mainly responsible for the constancy of the properties of the tumour is a superior viability of the stemline genotype. As long as the environment is one and the same, the stemline of such an old, well established tumour as the nearly tetraploid Ehrlich mouse ascites tumour, is probably superior to any deviating genotype that might appear. In the present investigation the transition to the new stemline was fortunately microscopically demonstrable by the decrease in chromosome number. However, nothing is known about the sequence of events or the mechanisms involved in this transition.

Radical environmental changes, such as those involved by transfer to a foreign species, surely will upset the above mentioned equilibrium. New genotypes may be able to compete favourably with those of the old stemline : a selection is started in the cell population.

Simultaneous mutations enhancing the viability of the new stemline in being may accumulate. A general increase in the rate of mutation is probably due to the change in environment imposed on the population by transfer of the tumour to a foreign host.

However, we do not know whether the disappearance of the four chromosomes from the original stemline implies a true loss of so much genetic material, or whether the loss is accompanied by translocations of the genic material in the chromosome

---

### EXPLANATION OF PLATES.

FIG. 1.—(*a*) Metaphase of Ehrlichs nearly tetraploid ascites cancer in mouse. Aceto-orcein staining. ×880. (*b*) Metaphase of the same cancer after serial transplantation through hamsters. Aceto-orcein staining. × 880. (*c*) Metaphase of " the adapted " cancer after retransplantation to, and serial transplantation through mice. Aceto-orcein staining. × 880.

FIG. 2.—(*a*) Metaphase of 2 *s* (octaploid) cell from Ehrlich nearly tetraploid ascites cancer after serial transplantation through hamsters. Aceto-orcein staining. × 880. (*b*) Metaphase with 2 large chromosomes in the same cancer after serial transplantation through hamsters. Aceto-orcein staining. × 880. (*c*) Metaphase cell of the same cancer after hamster passages : negative heteropyknosis. Aceto-orcein staining. × 880.

1a

1b

1c

Ising.

2a

2b

2c

Ising.

set so that the actual loss would comprise little more than four centromeres. The decrease in the quantity of chromatic substance due to the loss of the four chromosomes is too small to be demonstrable by DNA analysis.

It was interesting to note that the stemline number of 76 still persisted 3 months after the tumour had been transferred back to mice. This suggests that the chromosome number of this nearly tetraploid tumour is more liable to decrease than to increase.

FIG. 3.—(a) Idiogram analysis of Ehrlich nearly tetraploid ascites cancer in mouse. Colchicine treated. Camera lucida drawing. (b) Idiogram analysis of Ehrlich ascites cancer after 240 days' hamster passage. Colchicine treated. Camera lucida drawing.

In the first part of this paper it was reported that the tumour gained in virulence in the hamster. But we do not know with certainty whether this phenomenon is related to the chromosomal change. It should be recollected, however, that the occurrence of increasing virulence in most young tumours is regularly accompanied by a decrease in host specificity (Hauschka and Levan, 1953 ; Klein 1955), and by changes in chromosome number (Hauschka and Levan, 1953). It therefore appears reasonable to assume that the increased virulence of the tumour in hamsters was acquired in association with the development of the new stemline genotype, a development reflected by the change in the stemline chromosome number.

FIG. 4.

SUMMARY.

After having been carried for seven months in serial passages in hamster, the near-tetraploid Ehrlich mouse ascites tumour originally having a stemline chromosome number of 79–80 was shown to have shifted over to a stemline number of 76 chromosomes. After back-transfer for 30 passages in mouse hosts of the original strain the lower stemline number was still maintained. Two independent repetitions of this experiment have given similar results. No morphological difference in the chromosomes between the 76 and the 80-chromosome tumour type could be demonstrated.

### REFERENCES.

AHLSTRÖM, C. G. AND ISING, U.—(1955) *K. fysiogr. Sällsk. Lund Förh.*, **65**, No. 18.—(1955) *Acta path. microbiol. scand.*, **36**, 415.
HAUSCHKA, T.—(1952) *Cancer Res.*, **12**, 615.
*Idem* AND LEVAN, A.—(1953) *Exp. Cell Res.*, **4**, 457.
KLEIN, E.—(1955) *Ibid.*, **8**, 213.
LEVAN, A.—(1954) *Hereditas*, **40**, 1.
*Idem* AND HAUSCHKA, T.—(1952) *Ibid.*, **38**, 251.
TJIO, J. H. AND LEVAN, A.—(1954) *K. fysiogr. Sällsk. Lund Förh.*, **65**, No. 15.